# The promiscuous and highly mobile resistome of *Acinetobacter baumannii*

**DOI:** 10.1099/mgen.0.000762

**Published:** 2022-01-25

**Authors:** Ismael L Hernández-González, Valeria Mateo-Estrada, Santiago Castillo-Ramirez

**Affiliations:** ^1^​ Programa de Genómica Evolutiva, Centro de Ciencias Genómicas, Universidad Nacional Autónoma de México, Cuernavaca, Mexico

**Keywords:** antibiotic resistance, resistome, horizontal gene transfer, population genomics, genomic epidemiology, *A. baumannii*

## Abstract

Antimicrobial resistance (AR) is a major global threat to public health. Understanding the population dynamics of AR is critical to restrain and control this issue. However, no study has provided a global picture of the whole resistome of *Acinetobacter baumannii *, a very important nosocomial pathogen. Here we analyse 1450+ genomes (covering >40 countries and >4 decades) to infer the global population dynamics of the resistome of this species. We show that gene flow and horizontal transfer have driven the dissemination of AR genes in *A. baumannii *. We found considerable variation in AR gene content across lineages. Although the individual AR gene histories have been affected by recombination, the AR gene content has been shaped by the phylogeny. Furthermore, many AR genes have been transferred to other well-known pathogens, such as *Pseudomonas aeruginosa* or *Klebsiella pneumoniae *. Despite using this massive data set, we were not able to sample the whole diversity of AR genes, which suggests that this species has an open resistome. Our results highlight the high mobilization risk of AR genes between important pathogens. On a broader perspective, this study gives a framework for an emerging perspective (resistome-centric) on the genomic epidemiology (and surveillance) of bacterial pathogens.

## Data Summary

All the *Acinetobacter baumannii* genomes downloaded and used in this study are listed in Table S1 (available in the online version of this article). This table includes the accession number for each one of the genomes considered.

**Impact Statement**

Although *Acinetobacter baumannii* is a very important pathogen for which many genomes have been sequenced, there has been no effort made to understand the global resistome of this pathogen from a population genomics point of view. We conducted a population genomics analysis of almost 1500 *A *. *baumannii* genomes to analyse the transmission dynamics of the resistance genes in this species. Our data showed that gene flow and horizontal transfer have been the driving factors in the dissemination of AR genes in *A. baumannii *. There was substantial variation in resistance genes between and within the different lineages. Notably, many antibiotic resistance genes have been interchanged with other important pathogens, such as *Klebsiella pneumoniae* and *Pseudomonas aeruginosa *. Finally, our results implied that *A. baumannii* has an open resistome. Our study reveals the high mobilization of antibiotic resistance genes within the global population of *A. baumannii* and even with other common pathogens. Above all, this study embodies the idea of a resistome-centred genomic epidemiology of bacterial pathogens.

## Introduction

Antimicrobial resistance is a major global menace to humans all over the world. In this regard, the ESKAPE (* Enterococcus faecium *, *Staphylococcus aureus *, *Klebsiella pneumoniae *, *Acinetobacter baumannii *, *Pseudomonas aeruginosa* and *Enterobacter* species) group is a significant cause of deaths and burden disease in many countries [1]. This group of bacteria can easily acquire antimicrobial resistance genes (ARGs). In 2017 the World Health Organization issued a list of bacterial pathogens for which novel drugs are immediately required [2]. At the top of this list, with the highest priority status (priority 1: critical), was carbapenem-resistant *A. baumannii *. Notably, most *A. baumannii* hospital infections are caused by multidrug-resistant (MDR) isolates.

The resistome is the set of ARGs present in a given species or a particular environment [3]. Over the last decade, the resistome of some species and some ecological communities have been studied to an unprecedented detail due to the extensive amount of genetic information provided by genome sequencing. For instance, metagenomics and functional genomics studies have shown that ARGs are frequently found in many different environments [4–8]. These range from human-made environments, such as hospitals [5], to pristine and even isolated environments, such as isolated caves [7]. Furthermore, many ARGs are globally distributed and are rather diverse.

Genome sequencing has also been tremendously useful to better understand the biology of *A. baumannii *. Studies analysing the pangenome of this species have unveiled important aspects of its genetic variation [9] and its defence mechanisms [10, 11]. Additionally, population genomics and genomic surveillance studies have been very useful to study the geographical and temporal spreading of important lineages in this and other species [12–17]. However, most of these studies have used the core genome as the framework to understand the dynamics of the ARGs. Indeed, ARGs are often just mapped onto the core phylogeny. This strategy can be useful for rather clonal species, such as *Staphylococcus aureus *. Nonetheless, in species with high rates of gene content variation, such as *A. baumannii* [9], this strategy is bound to be problematic. Furthermore, ARGs are commonly found within mobile genetic elements. Hence, ARGs can be easily moved horizontally within and between species. In this regard, the potential transfer of ARGs between pathogens from different genera is especially concerning in hospital settings [6]. Therefore, to properly track the transmission dynamics of ARGs, population genomics studies focusing on the ARGs per se are required.

Over the last decade, and due to its clinical relevance, a lot of knowledge has been gained for *A. baumannii* through the genome sequencing of many hundred isolates [18]. However, despite the rapid accumulation of genomes, there has been a few studies that have tried to use all this information to analyse diverse aspects of *A. baummannii* [10, 19]. For instance, Mangas and colleagues focused on the distribution of CRISPR/Cas and plasmids in different lineages of the species [10], whereas Hamidian and Nigro focused on the distribution of some (but not all) the ARGs in *A. baummannii* [19]. Importantly, none of those studies paid attention to only including high-quality genomes. This is very relevant as this species has a very fluid genome with many mobile genetic elements; due to this, draft genomes often do not capture the whole repertoire of genes (ARGs included) present in a given isolate. Thus, to comprehensively study the population dynamics of the resistome of this species, high-quality genomes are required. Here we produce one of the most extensive views of the resistome of *A. baumannii* only including high-quality genomes. Our data set has almost 1500 high-quality genomes, covers 42 countries and four decades, and contains 149 lineages (sequence types). Our results showed that this is a very dynamic resistome, showing high levels of gene flow within the global population of *A. baumannii *. Furthermore, many ARGs are exchanged with other distantly related bacterial pathogens.

## Methods

### Genomes and quality check

Genomes sequences were downloaded in early March 2020 from the RefSeq NCBI Reference Sequence Database. Only genomes with reliable quality genome assembly status were considered; thus, only genomes showing an assembly level of ‘complete genome’, ‘chromosome’ and ‘scaffold’ were included in the final data set. We also used CheckM v1.0.11 [20] to evaluate the completeness and contamination of the genomes and only genomes showing 95 % (or higher) of completeness and equal or less than 5 % of contamination were considered for downstream analyses. We downloaded a total of 1554 but 82 genomes were discarded (these are listed in Table S3), as they did not pass the quality criteria or were not *A. baumannii* as per their ANI value (see below). These genomes come from 287 different Bioprojects (see Table S1). For consistency all the genomes were re-annotated with prokka v1.13 [21] and the final list of the genomes included is provided in Table S1. We also run a maximum-likelihood (ML) non-recombinant core phylogeny as we have done previously in a genomic epidemiology study of *A. baumannii* [22]. First, we defined the core genes and excluded all the core genes that had recombination signals (recombination was detected as stated below). A total of 50 non-recombinant genes were concatenated. On the concatenated alignment an ML phylogeny was run by means of RAxML v8.2.12 [23].

### Average nucleotide identity analysis and ST assignation

To make sure that all genomes belong to *A. baumannii* and to evaluate how similar they were, we conducted an average nucleotide identity (ANI) analysis using OrthoANI v 0.9 [24]. Using pairwise comparison, every genome was compared to the rest of the genomes and to the type strain ATCC 19606 (Biosample accession number SAMN13045090). We kept only the isolates that had an ANI value of 95 % or higher versus the type strain. We downloaded the allelic variants for profiles of all the ST described thus far for both the Oxford and the Pasteur schemes from the PubMLST database [25], accessed in late March 2020. Then, we used blastn (requiring 100 % identity) to assign an allelic profile to each genome considering both schemes. Given that the Oxford MLST scheme has some issues with paralogy in the locus *gdhB*, we processed the data as in [25] to discard the paralogous genes causing issues.

### Antibiotic resistance gene prediction and pangenome analysis

We employed the CARD database [26], accessed in late March 2020, to infer the ARGs in all the *A. baumannii* genomes. We used the Resistance Gene Identifier tool from CARD and only ‘Perfect’ and ‘Strict’ cases were considered but we also required a ≥90 % coverage between the query and the target. Antibiotic drug classes were also determined through CARD. Then, the ARGs were assigned to the different types of genome categories as follows: core genome, genes in 100 % of the isolates; soft core genome, genes between 95 % and less than 100 % of the isolates; shell genome, genes in less than 95 % but greater than 15 % of the isolates; and cloud genomes, genes in less than 15 % of the isolates. The ARGs were grouped into ARG families using the CARD classification.

### Recombination analysis, horizontal gene transfer with other species and accumulation curve analysis

Recombination analyses were run on all the ARG family groups that had at least three sequences and which shared at least 80 % of coverage among all the sequences. We used the PHIpack programme [27] that implements the ‘Pairwise Homoplasy Index’ test (PHI test) to detect recombination signals within the ARG families. To infer recent events of HGT between the ARGs in *A. baumannii* and other bacteria, we used blastn (with an e-value of 1e-50) to search these ARGs against the ‘nt’ database from the NCBI. Of note, as we wanted only very recent HGT events, we considered only those hits that were 100 % identical (both in the coverage and in the % of identity) to the query. As query could have identical hits from several species, we chose that species that had the highest number of identical hits. We used the Vegan R library version 2.5–7 [28] to run a rarefaction curve of the number of ARGs as a function of the number of genomes. We employed the ‘specaccum’ function for this, setting the method ‘rarefraction’. To establish whether the resistome was closed or open, we used the Heaps' law model; to that end we used the ‘heaps’ function in micropan version 2.2 [29], setting 100 000 permutations. The visualization tool Circos [30] was employed to illustrate the HGT cases between the ARGs in *A. baumannii* and the other three major bacteria contributors.

## Results

### The vast accessory resistome of *A. baumannii*

**Fig. 1.**
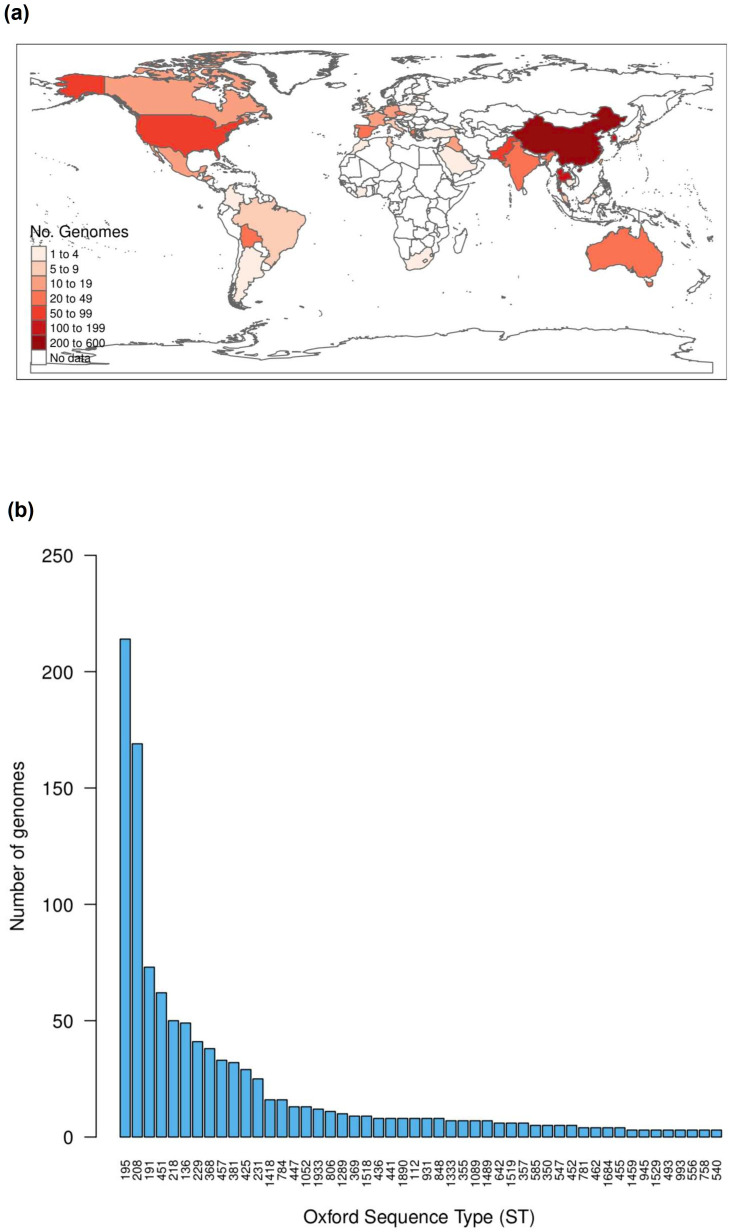
An extensive data set, covering many countries, years and lineages. (a) Countries from which the genomes were sampled, the colour key gives the number of genomes per region. (b) Number of genomes per ST under the Oxford scheme, only STs with two or more genomes are shown.

**Fig. 2.**
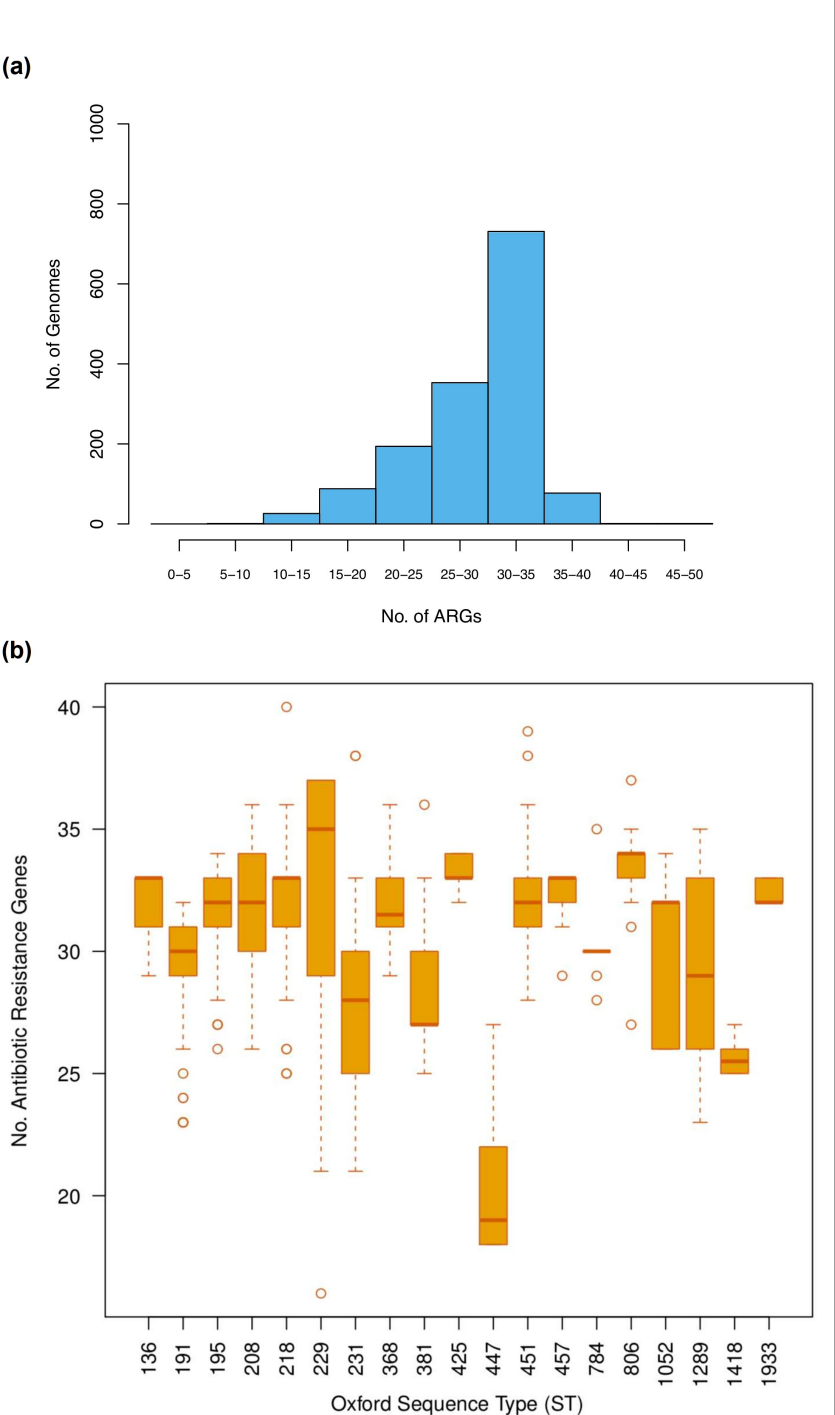
High variation of ARGs. (a) Histogram of the number of ARGs per genome. (b) Boxplots showing the variation in the number of ARGs within and between STs, only STs with at least ten genomes are shown.

To have the most comprehensive picture of the resistome of *A. baumannii *, we included as many genomes as possible; however, we did pay attention not to include lower-quality data. Thus, only genomes showing a high-quality assembly were downloaded (see Methods). Furthermore, we included only complete and uncontaminated genomes (completeness ≥95 % and contamination ≤5 %, see Methods). We also corroborated that all the genomes belonged to *A. baumannii* via an ANI analysis; where only isolates having ≥95 % identity with the type strain ATCC19606 were considered. We kept a total of 1472 genomes for downstream analyses (listed in Table S1). This is a considerably extensive data set for this species: it covered isolates from 42 different countries (Fig. 1a), a period of time spanning 76 years (four decades without outliers), and 149 sequence types (Fig. 1b). Importantly, this data set not was not biassed towards very closely related lineages, as the median ANI value (against the type strain) was 97.88 and the third quartile of all the ANI values was 97.93. Then, we used the Comprehensive Antibiotic Resistance Database [26] to catalogue and quantify the ARGs in the 1472 genomes. We found that the average number of ARGs per genome was 29.38; the frequency distribution histogram of ARGs per genome is presented in Fig. 2a. The ARGs were classified into 199 ARG families and these covered a wide range of drug classes (see Table 1 and S2). We noted that no single group was present in all the genomes (see Table 1). Twelve ARG families were present in more than 95 % of the genomes. These gene families correspond to the RND efflux pumps, well-known intrinsic resistance genes, and were present in most countries and in almost all the STs (see Table 1). We also found that 26 ARG families (13 %) were present in less than 95 % of the genomes but in more than 15 % of the genomes (see Table 1). Fig. S1 gives the distribution of the ARG families (grouped by type of drug class) on the core phylogeny, showing that many ARG families have undergone non-vertical transmission. Remarkably, almost all of these 38 most frequent ARG families were affected by recombination or horizontal gene transfer (see Table 1). However, the vast majority of the ARG families (161 groups, 81 %) were contained in less than 15 % of the genomes (seeTable S2). These ARG families were present in few countries and a few STs (see Table S2). Taken together, these results show that a considerable amount of ARGs is found within these genomes. Nonetheless, these ARGs do not belong to the core genome and are not strictly vertically transmitted. Notably, most of them are just present in less than 15 % of the strains.

**Table 1.** The most frequent ARGs found in the species

Gene family	No. genomes	No. countries	No. STs	Drug class
**abeS**	1470	42	149	macrolide
adeI	1469	42	149	Several classes
**abeM**	1469	42	149	fluoroquinolone
**adeK**	1464	42	149	Several classes
**adeG**	1461	42	146	fluoroquinolone, tetracycline
**adeH**	1460	42	148	fluoroquinolone, tetracycline
**adeF**	1460	42	148	fluoroquinolone, tetracycline
**adeJ** ^H^	1459	42	148	Several classes
**AmvA**	1456	42	148	macrolide
**parC**	1456	42	149	fluoroquinolone
**AbaQ**	1427	42	139	fluoroquinolone
**adeL**	1421	42	140	fluoroquinolone, tetracycline
**AbaF**	1389	41	132	fosfomycin
**adeB**	1377	41	132	glycylcycline, tetracycline
**adeA**	1346	42	131	glycylcycline, tetracycline
**adeR**	1332	41	130	glycylcycline, tetracycline
**adeS**	1323	41	128	glycylcycline, tetracycline
**gyrA**	1304	37	100	fluoroquinolone
**adeN**	1149	41	134	Several classes
**adeC**	1119	34	77	glycylcycline, tetracycline
OXA-23 ^H^	1111	29	80	cephalosporin
APH(3’)-Ib ^H^	1010	32	68	aminoglycoside
OXA-66	995	28	48	cephalosporin
APH(6)-Id ^H^	982	31	67	aminoglycoside
tetR ^H^	955	30	60	tetracycline
tet(B) ^H^	946	29	56	tetracycline
mphE ^H^	912	29	55	macrolide
msrE ^H^	905	29	55	Several classes
armA ^H^	895	22	44	aminoglycoside
sul2 ^H^	747	32	74	sulfonamide
TEM-1 ^H^	744	28	46	cephalosporin
APH(3’)-Ia ^H^	737	28	50	aminoglycoside
sul1 ^H^	602	27	52	sulfonamide
ADC-73	533	14	27	cephalosporin
ADC-30	422	19	27	cephalosporin
catB8 ^H^	329	13	22	phenicol
AAC(6’)-Ib9 ^H^	299	13	18	aminoglycoside
AAC(3)-Ia ^H^	231	20	34	aminoglycoside
APH(3’)-VIa ^H^	161	24	30	aminoglycoside

Information for the most frequent ARGs is provided. These ARGs were present in more than 100 genomes. The number of genomes, countries and ST in which every ARG was present is reported. In bold are those ARGs that had recombination signals and the superscript **^H^** means that identical allelic variants were found in other bacteria.

### High gene flow within the species and HGT with other pathogens

**Fig. 3.**
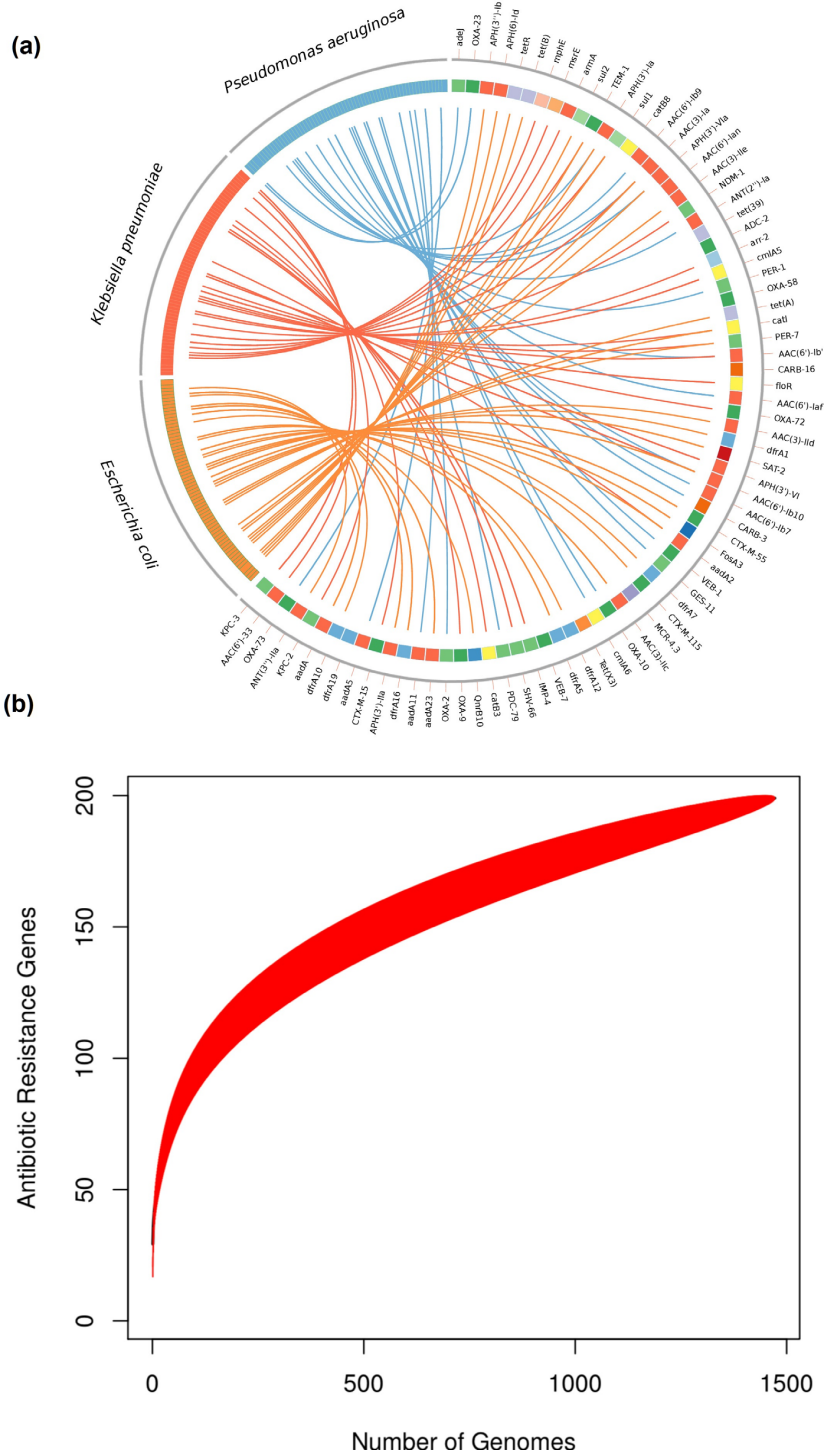
Horizontal transfer of ARGs and an open resistome. (a) Circos plot showing the cases of HGT between ARGs *A. baumannii* (the little squares from 1 to 7 clockwise) and other bacteria (on the left-hand side). Only bacteria (7 to 12 clockwise) involved in 97 % of the HGT cases are shown on the left-hand side. (b) Accumulation curve of the number of ARGs as a function of the number of genomes. The red area shows the confidence intervals.

The fact that most of the ARGs are just present in some of the genomes implies that ARGs are frequently lost and gained. In accordance with this, we recently showed that gene turnover was of paramount importance in a recently emerged lineage of this species [9]. To further explore this, we analyse ARG content variation within individual STs; we only considered those STs that had ten or more genomes per ST. This approach allowed us to analyse ARG variation over short timescales. If ARGs were to be only disseminated by clonal expansions (without HGT or gene loss), the amount of ARGs per genome would be the same for a given ST. Contrary to this, Fig. 2b clearly shows that there is a huge variation, not only within STs but also between STs (Kruskal–Wallis test, *P*-value<2.2e-16). While the ST that had most ARGs was ST229 with an average number of 32 ARGs per genome, the ST with the lowest mean number was ST447 (see Fig. 2b) with 20 ARGs per genome. We also conducted the same analysis but considering the Pasteur MLST scheme instead of the Oxford one (see Fig. S2); very similar patterns were observed, with a large variation both within and between STs (Kruskal–Wallis test, *P*-value<2.0e-16). This high variation in ARG content within STs implies that, even over short timescales, acquisitions and losses of ARGs are very common. Then we went on to look if any of these ARG families have been horizontally transferred very recently (see Methods). We noted that 78 ARG families (39%) had identical allelic variants in other bacteria (see Tables 1 and S2). Notably, most the HGT events were located in other nosocomial pathogens (see Table S2, Fig. 3a); *Klebsiella pneumoniae* and *Pseudomonas aeruginosa* are two of the most striking cases. Collectively, these results show that HGT (and gene loss) are of paramount importance not only within the species but also with other (nosocomial) bacterial species.

### An open resistome partially structured by the phylogeny

Although ARGs are subject to loss and gain processes, the presence of the ARGs could be structured by the phylogeny. This is because HGT between closely related lineages is likely to be more successful and also the more distantly related two lineages are, the higher the likelihood of gene loss. Thus, to explore this, we conducted pairwise comparisons of all the isolates correlating how similar they were in terms of their ARGs versus their ANI values. We used ANI as a proxy for the phylogenetic relationship of the isolates. To establish how similar the ARG profiles between the isolates were, we used the Jaccard index. A perfect correlation would imply that the gains and losses of ARGs are shaped by phylogeny. Although not very strong, Spearman’s correlation was significant (r^2^=0.57, *P*-value<2.2e-16). Thus, there is a correlation between ANI values and similarity in the ARG profiles, which implied that ARG content variation correlated with the phylogeny to some extent.

Finally, we wanted to establish if we were able to sample the whole diversity of ARGs in this species. Of note, given the very extensive nature of this data set in temporal and geographical terms, we would expect so. We ran an accumulation curve analysis to evaluate this (see Methods and Fig. 3b). This analysis showed that we have sampled much of the ARG family diversity. However, the curve did not level off and the slope is still very steep in the last section of the curve. Thus, the discovery rate of ARGs is still high after almost 1500 genomes and a lot more ARGs remain to be found.

To be completely sure about the openness of the resistome, we determine the value of the α parameter considering a Heaps' law model. Under this model an α value equal or lower than 1 denotes that the data are compatible with an open resistome; the value of the α parameter was 0.999 consistent with an open resistome. Taken together, these results imply that although gene flow is common within ARGs, ARG content variation within the isolates is structured to some extent by the phylogeny. Furthermore, this seems to be an open resistome.

## Discussion

Metagenomics studies over the last decade have been of paramount importance to characterize the resistome dynamics in different environments. However, reliable population genomic studies are still very much required to properly describe the transmission dynamics of ARGs both within and between bacterial species. A clear understanding of these transmission dynamics is essential for the effective use of antibiotics. Notably, this will provide useful knowledge to inform public health actions and infection control teams to establish better treatment options. However, there has not been a single study analysing the whole resistome of *A. baumannii* on a global scale. Here we gathered a very extensive – in terms of geographic and temporal components – dataset for this important nosocomial pathogen. Our analyses underscore a highly mobile and even promiscuous resistome. We found a considerable variation in ARGs profiles among the different lineages (STs). There are at least two contrasting forces that affects the distribution of the ARGs in *A. baumannii* populations. On the one hand, because most of these isolates are within clinical settings (under constant use of antibiotics) this will select for the presence of (at least some) ARGs. On the other hand, *A. baumannii* has defence mechanisms, for instance CRISPR/Cas systems or restriction-modification systems, that can impair the introduction of the mobile genetic elements harbouring the ARGs [10, 11]. In this regard, a recent study has shown that a group of *A. baumannii* having functional CRISPR/Cas systems have considerably fewer plasmids [10].

From a clinical standpoint, it is worth paying attention to the fact that many of the ARGs exchanged confer resistance to critically important antimicrobials for human health. For example, we found several ARGs conferring resistance against Aminoglycosides, which are frequently used to treat aerobic, Gram-negative infections in many parts of the world. We also detected transferable ARGs against Cephalosporins, employed to treat a wide variety of infections from both Gram-negative and Gram-positive bacteria. In connection with the previous point, our results have clear implications for the treatment against *A. baumannii* infections. For instance, we noted that several oxacillinases (OXAs), which confer resistance to Carbapenems (one of the last resort options to treat *A. baumannii* infections), were found in many STs and were even found in other pathogens. In this respect, it could happen that within the same locale (hospital) one would find several lineages of *A. baumannii *, some resistant and some susceptible. In this scenario, the resistant lineages can transfer the genetic resistance mechanism to the susceptible lineages; thus, rendering ineffective the potential antibiotic treatment. Importantly, this would not be limited just to lineages from *A. baumannii* but it could involve other pathogens, such as *K. pneumoniae *. Of note, the co-existence of several *A. baumannii* in a hospital is not unlikely. We have recently shown that different lineages of *A. baumannii* can co-exist in individual hospital settings [22, 31].

In some important bacterial pathogens, such as *K. pneumoniae *, MDR isolates (showing many ARGs) are frequently found in high-risk clones. Contrary to that, we found that in *A. baumannii* ARGs are well dispersed among the global population and clonal expansion does not seem to be the major force dispersing the ARGs. In this regard, previous studies have shown that gene-content variation is of paramount importance for this pathogen [9]. Importantly, even very recently emerged ARGs seem to have experienced HGT between different lineages of this species [16]. One of the most relevant findings of our study, from a clinical microbiology point of view, is that *A. baumannii* readily exchanges ARGs with other highly critical pathogens such as *K. pneumoniae* or *P. aeruginosa *. In this respect, a recent study also found many instances of HGT across MDR bacteria from different genera in a single hospital [32]. Hence, infection control and detection strategies considering this pathogen should be focused on the ARGs, rather than on particular lineages, to prevent the transmission of such genes. We think that the risk of ARGs across important nosocomial pathogens should be considered of paramount importance not only from a microbial genomics point of view but also from an infection control perspective. Finally, and related to the high amount of HGT with other pathogens, we observed that, notwithstanding the vast data set we used, our accumulation curve analysis implies that we did not sample the full diversity of ARGs within *A. baumannii *. Therefore, very likely this species has an open resistome; given the tendency of this species to acquire ARGs from other bacteria, an open resistome is not actually unexpected. Considering this, we have previously shown that this species has a very dynamic genome that easily acquires and losses genes [9], and where MLST schemes do not seem to work properly [33].

Our study has several limitations. First, it is not properly global, as we did not sample evenly the different regions of the world. Thus, our findings should be taken with caution. However, our study is by far one of the most extensive studies of the resistome of this species, not only geographically and temporally but also in terms of lineage diversity. Another limitation is that we underestimated the rate of HGT with other bacteria. This is because we only considered very recent HGT events, i.e. identical gene sequences in different bacteria. Clearly, the rate of HGT is bound to be considerably higher, if HGT events of different ages are to be included in the estimation. Another limitation of our study is that our analyses were based on a genetic definition of antibiotic resistance [34]. We acknowledged that further studies considering also microbiological and clinical definitions of antibiotic resistance are required to fully grasp the complex issue of antimicrobial drug resistance. Finally, future resistome studies might consider including not only *A. baumannii* but the whole set of closely related species conforming the *Acinetobacter calcoaceticus-Acinetobacter baumannii* complex (ACB complex). This is because this complex shows convoluted evolutionary relationships often prone to taxonomic misclassification of isolates [35]. Furthermore, several ARGs first described in *A. baumannii* have also been found in other species from the ACB complex. Thus, the inclusion of the different species within the ACB complex would allow for a more extensive portrayal of the underlaying population structure where the ARGs reside in.

Understanding the population dynamics of the ARGs is crucial for their tracking and surveillance, not only in the clinic but also in non-clinical settings [36]. In this regard, we recently show that environmental isolates of *A. baumannii* can be an important source of ARGs [37]. Without a doubt, the understanding of the transmission dynamics of ARGs can be used to implement ad hoc infection control actions within different health systems all over the world. Thus, similar studies are required for many other members of the ESKAPE group, or any other relevant human pathogen for that matter, to properly tackle the major problem of antimicrobial drug resistance.

## Supplementary Data

Supplementary material 1

Supplementary material 2

Supplementary material 3

Supplementary material 4

Supplementary material 5

## References

[1] De Oliveira DMP, Forde BM, Kidd TJ, Harris PNA, Schembri MA (2020). Antimicrobial Resistance in ESKAPE Pathogens. Clin Microbiol Rev.

[2] World Health Organization (2017). WHO publishes list of bacteria for which new antibiotics are urgently needed. www.who.int/news/item/27-02-2017-who-publishes-list-of-bacteria-for-which-new-antibiotics-are-urgently-needed.

[3] Waglechner N, Wright GD (2017). Antibiotic resistance: it’s bad, but why isn’t it worse?. BMC Biol.

[4] D’Costa VM, McGrann KM, Hughes DW, Wright GD (2006). Sampling the antibiotic resistome. Science.

[5] Evans DR, Griffith MP, Sundermann AJ, Shutt KA, Saul MI (2020). Systematic detection of horizontal gene transfer across genera among multidrug-resistant bacteria in a single hospital. Elife.

[6] Martínez JL, Coque TM, Baquero F (2015). What is a resistance gene? Ranking risk in resistomes. Nat Rev Microbiol.

[7] Pawlowski AC, Wang W, Koteva K, Barton HA, McArthur AG (2016). A diverse intrinsic antibiotic resistome from a cave bacterium. Nat Commun.

[8] Perry JA, Westman EL, Wright GD (2014). The antibiotic resistome: what’s new?. Curr Opin Microbiol.

[9] Graña-Miraglia L, Lozano LF, Velázquez C, Volkow-Fernández P, Pérez-Oseguera Á (2017). Rapid gene turnover as a significant source of genetic variation in a recently seeded population of a healthcare-associated pathogen. Front Microbiol.

[10] Mangas EL, Rubio A, Álvarez-Marín R, Labrador-Herrera G, Pachón J (2019). Pangenome of *Acinetobacter baumannii* uncovers two groups of genomes, one of them with genes involved in CRISPR/Cas defence systems associated with the absence of plasmids and exclusive genes for biofilm formation. Microb Genom.

[11] Tyumentseva M, Mikhaylova Y, Prelovskaya A, Tyumentsev A, Petrova L (2021). Genomic and phenotypic analysis of multidrug-resistant *Acinetobacter baumannii* clinical isolates carrying different types of CRISPR/Cas systems. Pathogens.

[12] Castillo-Ramírez S, Mateo-Estrada V, Gonzalez-Rocha G, Opazo-Capurro A (2020). Phylogeographical analyses and antibiotic resistance genes of *Acinetobacter johnsonii* highlight its clinical relevance. mSphere.

[13] Challagundla L, Reyes J, Rafiqullah I, Sordelli DO, Echaniz-Aviles G (2018). Phylogenomic classification and the evolution of clonal complex 5 methicillin-resistant *Staphylococcus aureus* in the Western Hemisphere. Front Microbiol.

[14] Frisch MB, Castillo-Ramírez S, Petit RA, Farley MM, Ray SM (2018). Invasive methicillin-resistant *Staphylococcus aureus* USA500 strains from the U.S. emerging infections program constitute three geographically distinct lineages. mSphere.

[15] Goldstone RJ, Smith DGE (2017). A population genomics approach to exploiting the accessory “resistome” of *Escherichia coli*. Microb Genom.

[16] Graña-Miraglia L, Evans BA, López-Jácome LE, Hernández-Durán M, Colín-Castro CA (2020). Origin of OXA-23 Variant OXA-239 from a recently emerged lineage of *Acinetobacter baumannii* international clone V. mSphere.

[17] López-Leal G, Zuniga-Moya JC, Castro-Jaimes S, Graña-Miraglia L, Pérez-Oseguera Á (2019). Unexplored genetic diversity of multidrug- and extremely drug-resistant *Acinetobacter baumannii* isolates from tertiary hospitals in Honduras. Microb Drug Resist.

[18] Evans BA, Kumar A, Castillo-Ramírez S (2021). Editorial: genomic basis of antibiotic resistance and virulence in acinetobacter. Front Microbiol.

[19] Hamidian M, Nigro SJ (2019). Emergence, molecular mechanisms and global spread of carbapenem-resistant *Acinetobacter baumannii*. Microb Genom.

[20] Parks DH, Imelfort M, Skennerton CT, Hugenholtz P, Tyson GW (2015). CheckM: assessing the quality of microbial genomes recovered from isolates, single cells, and metagenomes. Genome Res.

[21] Seemann T (2014). Prokka: rapid prokaryotic genome annotation. Bioinformatics.

[22] Mateo-Estrada V, Fernández-Vázquez JL, Moreno-Manjón J, Hernández-González IL, Rodríguez-Noriega E (2021). Accessory genomic epidemiology of cocirculating *Acinetobacter baumannii* clones. mSystems.

[23] Stamatakis A (2014). RAxML version 8: a tool for phylogenetic analysis and post-analysis of large phylogenies. Bioinformatics.

[24] Lee I, Ouk Kim Y, Park SC, Chun J (2016). OrthoANI: An improved algorithm and software for calculating average nucleotide identity. Int J Syst Evol Microbiol.

[25] Jolley KA, Bray JE, Maiden MCJ (2018). Open-access bacterial population genomics: BIGSdb software, the PubMLST.org website and their applications. Wellcome Open Res.

[26] Alcock BP, Raphenya AR, Lau TTY, Tsang KK, Bouchard M (2020). CARD 2020: antibiotic resistome surveillance with the comprehensive antibiotic resistance database. Nucleic Acids Res.

[27] Bruen TC, Philippe H, Bryant D (2006). A simple and robust statistical test for detecting the presence of recombination. Genetics.

[28] Oksanen J, Blanchet F, Kindt R, Legendre P, Minchin P (2018). vegan: Community Ecology Package. R package version.

[29] Snipen L, Liland KH (2015). micropan: an R-package for microbial pan-genomics. BMC Bioinformatics.

[30] Krzywinski M, Schein J, Birol I, Connors J, Gascoyne R (2009). Circos: an information aesthetic for comparative genomics. Genome Res.

[31] Graña-Miraglia L, Evans BA, López-Jácome LE, Hernández-Durán M, Colín-Castro CA (2020). Origin of OXA-23 Variant OXA-239 from a recently emerged lineage of *Acinetobacter baumannii* international clone V. mSphere.

[32] Evans DR, Griffith MP, Sundermann AJ, Shutt KA, Saul MI (2020). Systematic detection of horizontal gene transfer across genera among multidrug-resistant bacteria in a single hospital. Elife.

[33] Castillo-Ramírez S, Graña-Miraglia L (2019). Inaccurate multilocus sequence typing of *Acinetobacter baumannii*. Emerg Infect Dis.

[34] Courvalin P (2008). Predictable and unpredictable evolution of antibiotic resistance. J Intern Med.

[35] Mateo-Estrada V, Graña-Miraglia L, López-Leal G, Castillo-Ramírez S (2019). Phylogenomics reveals clear cases of misclassification and genus-wide phylogenetic markers for acinetobacter. Genome Biol Evol.

[36] Castillo-Ramírez S, Ghaly T, Gillings M (2021). Non-clinical settings - the understudied facet of antimicrobial drug resistance. Environ Microbiol.

[37] Hernández-González IL, Castillo-Ramírez S (2020). Antibiotic-resistant *Acinetobacter baumannii* is a One Health problem. The Lancet Microbe.

