# Supplementary material for: The promiscuous and highly mobile resistome of Acinetobacter baumannii
Source: Microb Genom. 2022 Jan 25;8(1):000762. doi: 10.1099/mgen.0.000762 (PMC8914355; doi:10.1099/mgen.0.000762)
Supplement: Supplementary material 1 [file mgen-8-0762-s001.pdf]

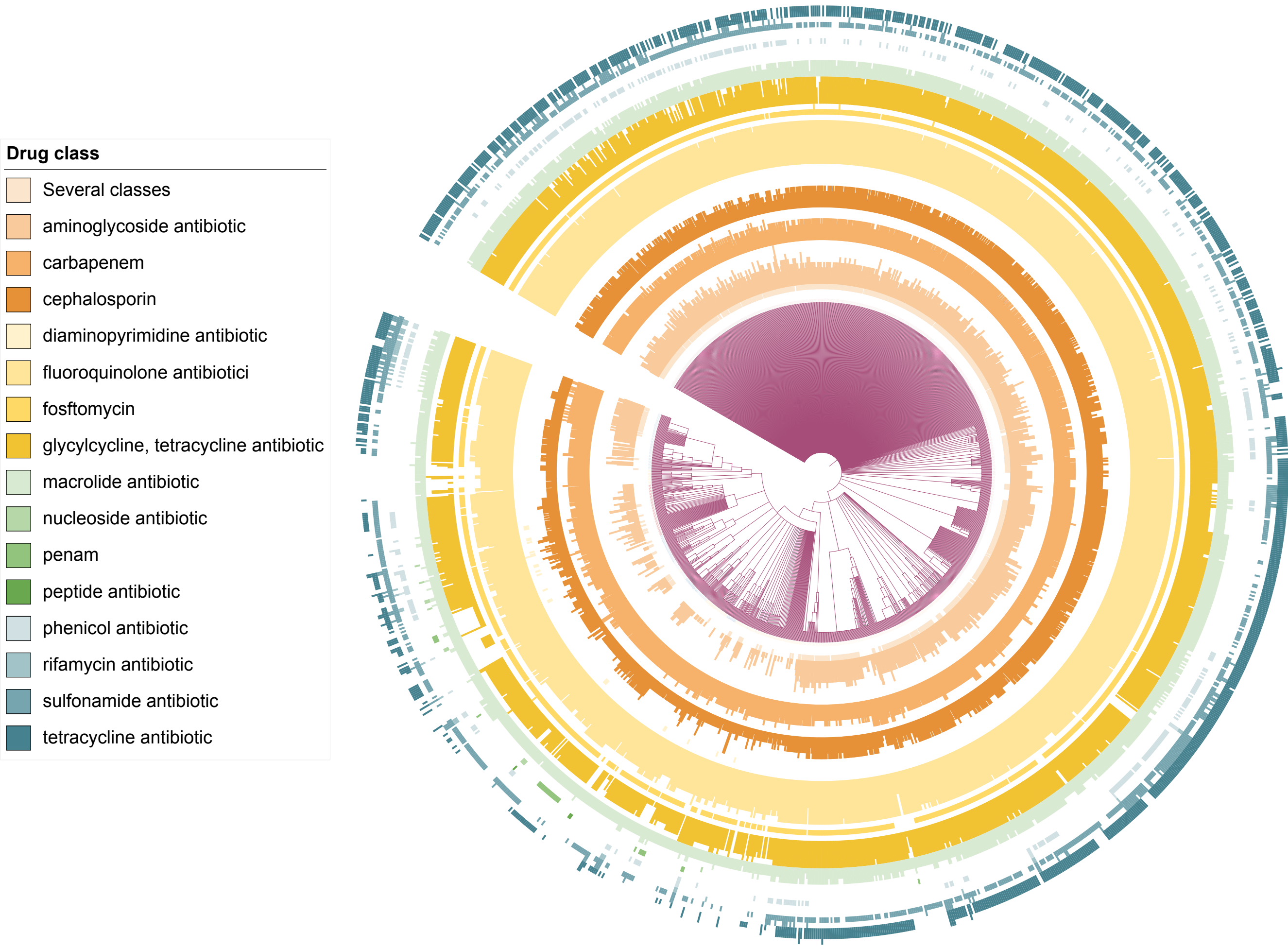


**Supplementary Figure 1**

ARG families (grouped by drug class) mapped on the non-recombinant core phylogeny.
