# Supplementary material for: The promiscuous and highly mobile resistome of Acinetobacter baumannii
Source: Microb Genom. 2022 Jan 25;8(1):000762. doi: 10.1099/mgen.0.000762 (PMC8914355; doi:10.1099/mgen.0.000762)
Supplement: Supplementary material 2 [file mgen-8-0762-s002.pdf]

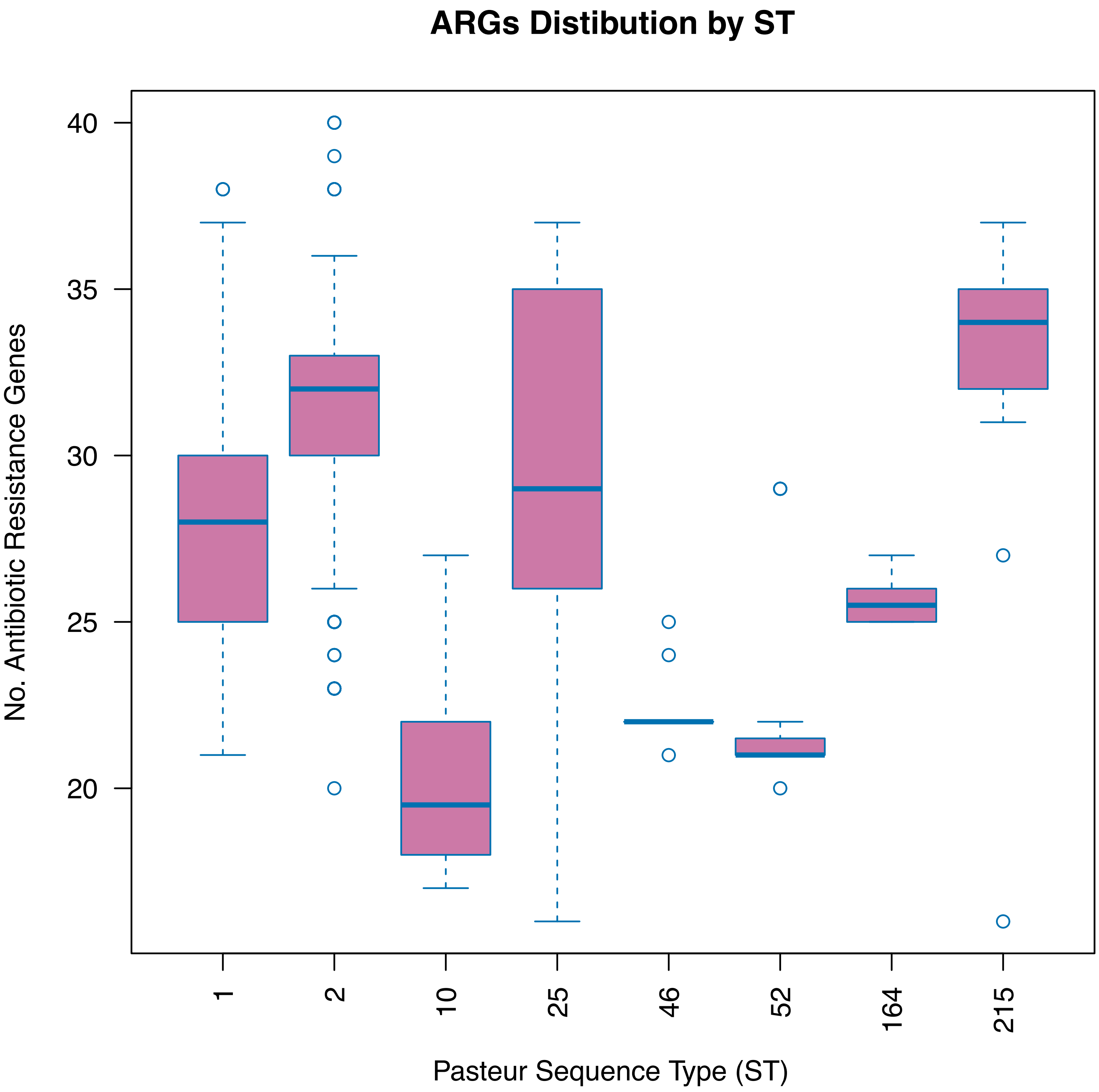

Supplementary Figure 2

Boxplots of the number of ARGs within and between STs, under the Pasteur scheme; only STs with at least 10 genomes are shown.
